# Blast phase myeloproliferative neoplasm: contemporary review and 2024 treatment algorithm

**DOI:** 10.1038/s41408-023-00878-8

**Published:** 2023-07-18

**Authors:** Ayalew Tefferi, Hassan Alkhateeb, Naseema Gangat

**Affiliations:** Division of Hematology, Department of Medicine, Mayo Clinic, Rochester, Minnesota USA

**Keywords:** Myeloproliferative disease, Myeloproliferative disease

## Abstract

Leukemic transformation in myeloproliferative neoplasms (MPN), also referred to as “blast-phase MPN”, is the most feared disease complication, with incidence estimates of 1–4% for essential thrombocythemia, 3–7% for polycythemia vera, and 9–13% for primary myelofibrosis. Diagnosis of MPN-BP requires the presence of ≥20% circulating or bone marrow blasts; a lower level of excess blasts (10–19%) constitutes “accelerated phase” disease (MPN-AP). Neither “intensive” nor “less intensive” chemotherapy, by itself, secures long-term survival in MPN-BP. Large-scale retrospective series have consistently shown a dismal prognosis in MPN-BP, with 1- and 3-year survival estimates of <20% and <5%, respectively. Allogeneic hematopoietic stem cell transplant (AHSCT) offers the possibility of a >30% 3-year survival rate and should be pursued, ideally, while the patient is still in chronic phase disease. The value of pre-transplant bridging chemotherapy is uncertain in MPN-AP while it is advised in MPN-BP; in this regard, we currently favor combination chemotherapy with venetoclax (Ven) and hypomethylating agent (HMA); response is more likely in the absence of complex/monosomal karyotype and presence of *TET2* mutation. Furthermore, in the presence of an *IDH* mutation, the use of IDH inhibitors, either alone or in combination with Ven-HMA, can be considered. Pre-transplant clearance of excess blasts is desired but not mandated; in this regard, additional salvage chemotherapy is more likely to compromise transplant eligibility rather than improve post-transplant survival. Controlled studies are needed to determine the optimal pre- and post-transplant measures that target transplant-associated morbidity and post-transplant relapse.

## Introduction

*JAK2* mutation-prevalent myeloproliferative neoplasms (MPN) include primary myelofibrosis (PMF), polycythemia vera (PV), essential thrombocythemia (ET), and MPN, unclassifiable (MPN-U) [1, 2]. Each one of these entities carries a risk of disease transformation into acute myeloid leukemia (AML), formally designated as blast phase disease (MPN-BP) [2]. Diagnosis of MPN-BP requires the presence of ≥20% circulating or bone marrow blasts, while a blast count of 10–19% constitutes “accelerated phase” disease (MPN-AP) [1, 3]. In an international study of 1581 patients with MPN, 826 were from the Mayo Clinic and followed for a median of over 17 years for living patients; transition into MPN-BP was documented in 4.1% of patients with ET, 6.7% PV, and 12.7% PMF [4]. The study also included 755 Italian patients followed for a shorter period of time and with corresponding leukemic transformation rates of 1.4%, 3.2%, and 11.8% [4]. Calculation of leukemic-free survival in the Mayo Clinic cohort of the particular study [4] favored ET over both PMF and in PV and, in the Italian cohort, PV and ET over PMF [4]. In a more recent study of over 3000 patients with MPN, reported rates of transition into MPN-BP from ET, PV, or PMF were 2.6%, 3.9%, and 9.3%, respectively, after a median follow-up of 9.9, 8.2, and 3.2 years [5].

In PMF, risk factors for leukemic transformation include *IDH1*, *IDH2*, *SRSF2* or *ASXL1* mutation, high risk/unfavorable karyotype, circulating blasts ≥3%, age >70 years, moderate/severe anemia, and thrombocytopenia [6–10]; a risk model based on these risk factors distinguished a high-risk group with BP-MPN incidence of 57%, intermediate-risk 17% and low-risk 8% [10]. Risk factors for leukemic transformation in PV include *SRSF2, IDH2*, or *RUNX1* mutation, older age, leukocytosis, and abnormal karyotype [11–13], and in ET *TP53*, *SRSF2*, *EZH2, U2AF1*, or *RUNX1* mutation, del(20q) karyotype, prefibrotic morphology, thrombosis, extreme thrombocytosis, and anemia [11, 13–16]. It is important to be familiar with these risk factors and closely monitor the individual patient with chronic phase MPN in order to intervene with allogeneic hematopoietic stem cell transplant (AHSCT) before disease transformation into overt MPN-BP [17].

### Natural history of blast phase myeloproliferative neoplasm

The two largest studies in MPN-BP were led by Mayo Clinic investigators and included patients diagnosed before [18] and after [19] the FDA approval date of ruxolitinib (2011). The first study [18] included a total of 410 patients recruited from the Mayo Clinic (*N* = 248; median age 67 years; 65% males) and the University of Florence, Italy (*N* = 162; median age 69 years; 57% males) [Mayo-AGIMM study]. In the Mayo Clinic patient cohort of the particular study [18], the antecedent MPN subtype was PMF in 118 (48%) patients, PV in 60 (24%), and ET in 70 (28%); among the 60 patients with post-PV MPN-BP, 32 (53%) experienced leukemic transformation without transitioning through fibrotic progression while the corresponding rate for post-ET MPN-BP was 56% [18]. Morphologic variants of MPN-BP in the Mayo Clinic cohort included AML-M7 in 7%, AML-M6 1%, AML with recurrent favorable cytogenetic abnormalities 1%, and myeloid sarcoma 3%. Cytogenetic information was available in 172 cases in the Mayo Clinic cohort, out of which 140 (81%) were reported abnormal, including 56 (40%) with “high risk” abnormalities including monosomal karyotype, monosomy, inv(3)(q21.3q26.2)/t(3;3)(q21.3;q26.2), and i(17)(q10); cytogenetic profile and prevalence of *JAK2*V617F mutation were similar between post-PMF and post-PV/ET MPN-BP [18].

In the aforementioned Mayo Clinic patient cohort (*N* = 248) from the Mayo-AGIMM study [18], 96% of the patients were dead after a median follow-up of 3.6 months with 1-, 3- and 5-year survival rates of 17, 6, and 4%, respectively [18]; treatment included supportive care (*N* = 121; 49%), chemotherapy (*N* = 103; 42%) with (*N* = 24) or without (*N* = 79) achieving complete remission (CR; 35% rate) or CR with incomplete count recovery (CRi; 24% rate), and AHSCT (*N* = 24;10%); the 1- and 3-year survival rates were 66% and 32% for AHSCT, 37% and 19% for patients achieving CR/CRi but were not transplanted, and 8% and 1% in the absence of both AHSCT and CR/CRi, respectively [18]; survival trends were significantly better for patients diagnosed in the year 2000 and afterward, but with no additional improvement since then; median survivals prior to the year 2000 vs. in the years 2000–2009 vs. in 2010–2018 were 2.3, 3.5, and 4.9 months, respectively [18]. Favorable risk factors for survival in the particular study were receiving AHSCT, achieving CR/CRi, absence of high-risk karyotype, and absence of thrombocytopenia (platelet count < 100 × 10^9^/L. Similar observations were made in the Italian cohort of the Mayo-AGIMM study [18].

The more recent Mayo Clinic study in MPN-BP included 103 patients (median age 70 years, range 37–89; 52% males) diagnosed after the approval date of ruxolitinib in the period between 2011 and 2021 [19]. In this particular study, MPN variant prior to transformation to MPN-BP was PMF in 35% and post-PV/ET MF in 65%; MPN treatment prior to leukemic transformation included ruxolitinib ± other JAK2 inhibitors in 32 (31%) patients while the remaining 71 cases received other cytoreductive drugs or supportive care; at the time of leukemic transformation, karyotype was available in 97 patients and revealed monosomal karyotype or monosomy 7 in 35 (36%), complex karyotype, non-monosomal in 18 (19%), normal karyotype in 17 (18%) and other abnormalities in 27 (28%); driver mutation distribution was *JAK2* 67%, *CALR* 7%, *MPL* 6% and triple-negative 1%; neither the karyotype nor the driver mutation profile was affected by prior exposure to ruxolitinib [19]. The study also included information on other mutations, the most frequent being *ASXL1* (40%), *TP53* (33%), *TET2* (18%), *FLT3* (18%), SRSF2 (16%), *EZH2* (16%), *DNMT3A* (16%), *IDH1* (16%), *RUNX1* (11%), and *NRAS* (9%); frequency of *SRSF2* mutation was significantly higher in patients previously exposed to ruxolitinib (16% vs. 3%) [19].

Observations from the abovementioned ruxolitinib era study [19] were not too dissimilar from the earlier study [18], also discussed above. First-line MPN-BP therapy in the ruxolitinib era study (*n* = 103) included intensive chemotherapy (*n* = 35; 35%), hypomethylating agents (HMA) with (*n* = 12; 12%) or without (*n* = 21; 21%) venetoclax, other drugs (*n* = 6; 6%) or supportive care (*n* = 25; 25%); reported CR/CRi were significantly lower at 15% among 71 evaluable cases, and at the time of the study report, 93% of the patients had died while 11 patients had undergone AHSCT; only 7 patients were censored alive, with documented AHSCT in 5; of note, 4 of the 5 survivors post-transplant had persistent bone marrow blasts (8–16%) at time of their transplant. Similar to the study before it, AHSCT and achieving CR/CRi had a positive effect on survival, while older age, complex/monosomal karyotype, thrombocytopenia, and, interestingly, prior exposure to ruxolitinib were detrimental to survival [19]. Table 1 summarizes the observations from other studies (some including patients with MPN-AP) [20–24], whose findings were not too dissimilar from those published by Mayo Clinic investigators, as elaborated above; in short, even if one was to consider the most recent studies, median survival remained <6 months with treatment-induced CR/CRi providing a short-term survival advantage, which might further be reinforced by AHSCT (Table 1).

**Table 1:** Summary of retrospective studies on patients with blast phase myeloproliferative neoplasms (some studies have included patients with accelerated phase disease) (*References cited in the text*).

Study	MPN-BP type	Driver mutation	Karyotype	Treatment *N* (%)	Response	AHSCT *N* (%)	Survival	Risk factors	Impact of treatment on survival
Tefferi et al. *Leukemia 2018* Mayo cohort *N* = 248 Median age 67 years	Post-PMF (*N* = 118) Post-ET/PV (*N* = 130)	*JAK2* (68%)	Abnormal (81%) Adverse (23%)	Intensive chemo 69 (28) HMA 26 (11) Other agents 30 (12)	Intensive chemo 59% CR/CRi HMA 3% CR/CRi	24 (10)	Median 3.6 months 1/3/5-year 17/6/4%	Karyotype Thrombocytopenia age >65 years Transfusions	1/3/5-year: AHSCT 66/32/10% CR/CRi without AHSCT 37/19/13% No CR/CRi No AHSCT 8/1/1%
Tefferi et al. *Leukemia 2018* Florence cohort *N* = 162 Median age 69 years	Post-PMF (*N* = 70) Post-ET/PV (*N* = 92)	*JAK2* (61%)	Abnormal (61%)	Intensive chemo 48 (30)	Intensive chemo 35.4% CR/CRi	25 (15)	Median 3.6 months 1/3-year 25/11%	-	1/3-year: AHSCT in CR/CRi 69/30% AHSCT without CR/CRi 62/38%
Abdelmagid et al. *Haematologica 2023* *N* = 103 Median age 70 years	Post-PMF (*N* = 35) Post-ET/PV (*N* = 64)	*JAK2* (67%)	Abnormal (83%)	Intensive chemo 35 (35) HMA 21 (21) HMA+venetoclax 12 (12)	Intensive chemo 15% CR/CRi	11 (11)	Median 6.7 months No ruxolitinib exposure Median 3.7 months with ruxolitinib exposure	Age >65 years, Karyotype Thrombocytopenia Ruxolitinib	3-year/Median: Intensive chemo without AHSCT 3%/4.7 months Less intensive chemo without AHSCT 3%/5.4 months
Mesa et al. *Blood 2005* *N* = 91 Median age 66 years	Post-PMF (*N* = 49) Post-ET/PV (*N* = 42)	-	Abnormal (91%) Adverse (61%)	Intensive chemo 24 (26) Less intensive chemo 19 (21)	Intensive chemo 0% CR 41% reverted to Chronic phase	-	Median 2.7 months	-	Median: Intensive chemo 3.9 months Less intensive chemo 2.9 months
Tokumori et al. *Clinical Lymphoma* *Myeloma & Leukemia* *2022* *N* = 75 Median age 66 years	-	*JAK2* (71%) *MPL* (14%)	-	Intensive chemo 28 (37) HMA based, 28 (37)	Intensive chemo 70% CR/CRi HMA based 29% CR/CRi	15 (20)	Median 4.8 months	No CR/CRi No AHSCT	Median: Intensive chemo 11.4 months Less intensive chemo HMA 4.7 months AHSCT not reached
Mollard et al. *Leuk Lymphoma 2018* *N* = 122 Median age 66 years AP-MPN, *N* = 48 BP-MPN, *N* = 74	Post-PMF (*N* = 30) Post-ET/PV (*N* = 92)	*JAK2* (61%) *CALR* (7%) *MPL* (3%)	Adverse (61%)	Intensive chemo 13 (11) HMA 24 (20)	Intensive chemo 62% CR/CRi HMA 33% CR/CRi	11 (15)	Median 4 months	-	Median: Intensive chemo 10.2 months HMA 9 months AHSCT 19.4 months
Tam et al. *Blood 2008* *N* = 74 Median age 64 years	Post-PMF (*N* = 36) Post-ET/PV (*N* = 32) Post-MPN-U (*N* = 6)	*JAK2* (43%)	Abnormal (72%) Adverse (48%)	Intensive chemo 41 (55) Less intensive chemo 12 (16)	Intensive chemo 46% CR/CRi Less intensive chemo 0% CR/CRi	11 (15)	Median 5 months	PS Splenectomy Karyotype	Median: Intensive chemo without AHSCT 6 months Less intensive chemo without AHSCT 7 months CR/CRi Without AHSCT 13 months
Patel et al. *Blood 2022* *N* = 80 Median age 69 years AP-MPN, *N* = 16 BP-MPN, *N* = 64	Post-PMF (*N* = 16) Post-ET/PV (*N* = 33) Post-MPN-U (*N* = 21)	*JAK2* (66%) *CALR* (14%) *MPL* (10%)	-	Intensive chemo 32 (40) HMA 16 (20) HMA+venetoclax 23 (29) IDH inhibitor 3 (4)	Intensive chemo 28% CR/CRi HMA 19% CR/CRi HMA+venetoclax 30% CR/CRi IDH inhibitor 33% CR/CRi	21 (26)	Median 8.8 months	-	Median: Intensive chemo 7 months HMA+ venetoclax 7.2 months HMA 13.2 months AHSCT 16 months
Lancman et al. *Leuk Res 2018* *N* = 57 Median age 68 years	Post-PMF (*N* = 13) Post-ET/PV (*N* = 39)	*JAK2* (64%)	Adverse (63%)	Intensive chemo 12 (21) HMA 27 (47) Other agents 6 (11)	Intensive chemo 67% CR/CRi HMA 15% CR/CRi	19 (33)	Median 5.8 months 2-year 28%	Transfusions	Median: Intensive chemo Not reached HMA 6.7 months AHSCT Not reached

*PMF* primary myelofibrosis, *ET* essential thrombocythemia, *PV* polycythemia vera, *AML* acute myeloid leukemia, *chemo* chemotherapy, *PS* performance status, *HMA* hypomethylating agent, *AHSCT* allogeneic hematopoietic stem cell transplant, *CR* complete remission, *CRi* complete remission with incomplete count recovery, *CMML* chronic myelomonocytic leukemia, *MPN-U* myeloproliferative neoplasm unclassified, *IDH* isocitrate dehydrogenase.

### Recent reports of less intensive induction chemotherapy in blast phase myeloproliferative neoplasm

At the present time, there are no controlled studies that could inform the optimal induction chemotherapy for patients with MPN-BP. Instead, several retrospective or single-arm prospective studies are available for review, and most included hypomethylating agent (HMA)-based combination therapy, as outlined in Table 2. The most prominent in this regard is the study by Gangat et al., which retrospectively examined the value of HMA combined with venetoclax (HMA-Ven) [25–27]. In the most recent account of the latter study [25], 47 (median age 71 years; range 46–84) patients with MPN-BP were included and received azacitidine 75 mg/m^2^ days 1–7 or decitabine 20 mg/m^2^ days 1–5 along with Ven 200 mg (range, 100–400 mg) daily administered for a median of 3 cycles (range, 1–9 cycles). The major side effect of HMA-Ven was severe and sometimes protracted pancytopenia that occurred in 62% of the study patients and was associated with neutropenic fever in 47% of the cases.

**Table 2:** Hypomethylating agent (HMA) combination therapy in patients with accelerated or blast phase myeloproliferative neoplasms (MPN-AP/BP) (*References cited in the text*).

Study	Study design MPN type	Treatment regimen	Response rates (duration)	Toxicity	Median survival
**HMA + Ruxolitinib**					
Mascarenhas et al. *Blood Advances 2020* *N* = 25	Phase 2 MPN-AP (*n* = 10) MPN-BP (*n* = 15)	HMA+ruxolitinib	CR 0% CRi 8% PR 36% ORR 44% (3.4 months)	Grade 3/4 Febrile neutropenia 28% Pneumonia 24% Neutropenia 16% Anemia 16% Bone pain 8% Thrombocytopenia 8%	9.5 months
Rampal et al. *Blood Advances 2018* *N* = 21	Phase 1 MPN-AP (*n* = 8) MPN-BP (*n* = 13)	HMA+ruxolitinib	CRi 24% PR 29% ORR 53%	Grade 3/4 Febrile neutropenia 33% Pneumonia 29% Thrombocytopenia 19% Anemia 14% Sepsis 14%	7.9 months
Bose et al. *Leukemia 2020* *N* = 14 (Phase 1) *N* = 18 (Phase 2)	Phase 1/2 MPN-BP	HMA+ruxolitinib	CR 7% CRi 34% PR 3% ORR 45% (1.7 months)	Grade 1/2 Fatigue Pruritus Diarrhea Nausea	6.2 months
Drummond et al. *Blood 2020* *N* = 34	Phase 1/2 MPN-AP (*n* = 19) MPN-BP (*n* = 15)	HMA+ruxolitinib	MPN-AP CR 5% Marrow CR 21% PR 5% (10.7 months) MPN-BP PR 27% (6.6 months)	-	1-year survival 42%
**HMA + Venetoclax**					
Gangat et al. *Haematologica 2022* *N* = 47	Retrospective MPN-BP (*N* = 47) ND (*n* = 32) Relapsed (*n* = 15)	HMA+Ven	CR 26% CRi 17% PR 11% ORR 53% (5 months)	Pancytopenia 62% Neutropenic fever 47% Major hemorrhage 2% Gastrointestinal 11%	7 months 1-year survival 28%
Masarova et al. *Blood Advances 2021* *N* = 31	Retrospective MPN-BP ND (*n* = 14) Relapsed (*n* = 17)	HMA+Ven Ven+other *Cladribine* *LDAC* *IDHi* *CPX-351* *CLIA* *FLAG(+/-ida)*	CR 10% CRi 10% PR 3% ORR 23% Relapsed pts 0%	≥Grade 3 infection 84% Severe hemorrhage 45% CNS hemorrhage 19%	4 months 1-year survival 16%
King et al. *AJH 2021* *N* = 27	Retrospective MPN-BP (*n* = 21) ND (*n* = 8) MPN-AP (*n* = 6)	HMA+Ven LDAC+Ven	MPN-BP CR 24% PR 19% ORR 52% (2.9 months) MPN-AP CR 50% 1.8 months	MPN-BP Infection 28% Grade 3 hemorrhage 19% MPN-AP Neutropenic fever 50%	6 months
Tremblay et al. *Leuk Res 2020* *N* = 9	Retrospective MPN-BP (*n* = 8) MPN-AP (*n* = 1) ND (*n* = 2)	HMA+Ven	CR 11% CRi 22%	≥Grade 3 Infection 78% Hemorrhage 56%	4.2 months

*CR* complete remission, *CRi* complete remission with incomplete count recovery, *PR* partial remission, *ORR* overall response rate, *HMA* hypomethylating agent, *ND* newly diagnosed, *Ven* venetoclax, *LDAC* low dose cytarabine.

Treatment response to HMA-Ven in the above study [25] was 26% CR, 17% CRi, and 11% partial response resulting in 53% overall response; it should be noted that 10 of the patients with CR/CRi harbored residual morphological features of MPN; median time to CR was 1.7 months (range; 1–7 months), with median response duration of 5 months (range, 0.4–35 months). Importantly, 7 of 13 (54%) transplant-eligible patients that achieved CR/CRi were successfully bridged to AHSCT. Additional details from the particular study [25] revealed similar CR/CRi rates between patients who received HMA-Ven upfront or in the relapsed setting, with azacitidine or decitabine, or with or without prior HMA exposure; CR/CRi rates were also not affected by MPN driver mutation status or presence or absence of *TP53* (41% vs. 44%), *ASXL1* (47% vs. 41%), *IDH1/2* (50% vs. 41%), or K/NRAS (20% vs. 46%) mutations. However, CR/CRi was significantly higher in the presence of *TET2* mutation (70% vs. 35%) and absence of complex/monosomal karyotype (60% vs. 29%), antecedent PV (55% vs. 19%), or thrombocytopenia (*p* = 0.10) [25]. Median survival in the study was 7 months (range; 1–37 months) with 1/2/3-year survival rates of 28%/15%/15% and longer in the presence of AHSCT (11 vs. 6 months; 1/2/3-year survival, 46%/30%/30% in transplanted vs. 25%/16%/0% in non-transplanted cases); post-transplant survival was adversely affected by complex karyotype and *N/KRAS* mutations [25].

Other retrospective studies with HMA-Ven therapy for MPN-BP are difficult to interpret because of the small sample size [28], the inclusion of patients with MPN-AP [29], or the utilization of various regimens in combination with Ven (Table 2) [30]. Single-arm prospective studies have examined the value of ruxolitinib combined with HMA in MPN-BP/MPN-AP (Table 2) [31–34]. In a phase 2 study of ruxolitinib plus decitabine in patients with either MPN-BP or MPN-AP, the overall response rate was 44% (CR/CRi/partial remission (PR) of 0%, 8%, and 36%, respectively) per the modified Cheson criteria [32]. Others have reported higher CR/CRi rates (24–41%) with HMA + ruxolitinib, but the reports are confounded by the inclusion of patients with MPN-AP in some of these studies (Table 2) [31, 33]. It is important to note the substantial number of treatment-emergent side effects associated with both ruxolitinib and venetoclax combinations with HMA, as detailed in Table 2. Taken together, it is evident that there is no consistency of observations across different studies, partly related to patient selection and the use of variable response criteria. What is evident, however, is the suboptimal CR rates and their short duration, regardless of specific treatment regimens and the inadequacy of chemotherapy as a whole to secure long-term survival.

In general, although not tested in a prospective controlled setting, HMA-based combination therapy is believed to be superior to HMA alone [35], as has been the case with AML [36]. It is, however, not clear if treatment approaches that are more intensive than Ven- or HMA-based combinations would result in higher or more durable responses and if they make a difference in regard to post-transplant survival. We suspect that intensive AML-like induction therapy might result in higher CR rates, but this has to be confirmed in a controlled setting; in the aforementioned Mayo Clinic study [18], the respective CR rates for AML-like induction chemotherapy, HMA, and other investigational drugs were 35, 4, and 3%; an additional 24% of patients who received AML-like induction chemotherapy achieved CRi, which was not observed in patients treated with HMA. In other words, the likelihood of obtaining CR/CRi was 59% following AML-like induction chemotherapy vs. <5% with HMA or other agents. Whether or not intensive chemotherapy results in higher CR/CRi rates, compared to Ven-HMA, requires controlled examination but single-arm studies with the latter have reported CR/CRi rates (20–43%; Table 2) [25, 28–30] that are significantly higher than seen with historical controls treated with HMA alone.

Other drugs used for the treatment of MPN-BP/AP include CPX-351 and IDH1/2 inhibitors [37–39]. It is to be noted that the original CPX-351 studies in AML did not include patients with MPN-BP [40]. A recent retrospective study of 12 CPX-351-treated patients with MPN-BP reported a CR (ELN criteria) rate of 25% that was not noticeably different than those observed with HMA-Ven (CR 26% per ELN criteria) [25, 39]; of note, some patients failing treatment with CPX-351 were successfully salvaged with HMA-Ven and transitioned to AHSCT [39]. Our observations with CPX-351 in MPN-BP are consistent with a recent report showing similar outcomes of AML patients treated with CPX-351 vs. HMA-Ven [41]. Current experiences with IDH inhibitors in MPN-BP/AP, used alone or in combination with other chemotherapy, are promising and worthy of additional investigation [37, 38]. As a background, *IDH1/2* mutations occur in 2–4% of MF patients in chronic or accelerated phase disease [42] and approximately 19% in MPN-BP [43]. IDH inhibitors as monotherapy or combined with other chemotherapy have shown impressive activity in IDH-mutated AML, both in the upfront and relapsed/refractory setting [44]; reported overall (ORR) and complete (CR) response rates with ivosidenib [45] or enasidenib [46] monotherapy were approximately 40% and 20%, respectively, and in combination with chemotherapy ranged from 63 t% to 89% for ORR and 47% to 68% for CR [44].

A study of 12 patients with IDH1/2-mutated MPN-BP used combination therapy with IDH1/2 inhibitors ± HMA ± ruxolitinib ± other drugs [38]; differentiation syndrome, a characteristic drug toxicity, was reported in 5 (42%) patients; 3 (43%) of 7 patients treated in the frontline setting achieved CR after receiving enasidenib (IDH2 inhibitor) + azacytidine + ruxolitinib; or ivosidenib (IDH1 inhibitor) + venetoclax; or enasidenib + “7 + 3” AML-like induction therapy, and all three cleared their mutant *IDH*; one of the 3 patients transitioned into AHSCT while one was in sustained clinical and molecular response for over 2 years [38]. In this particular study, CR was not observed in any of the patients receiving IDH inhibitor monotherapy [38]. In another more informative study of 8 patients with *IDH2*-mutated MPN-BP/AP (bone marrow blasts 10–80%) [37], 6 received enasidenib in the upfront and 2 relapsed/refractory setting; among the former, 5 patients received enasidenib monotherapy with one (20%) achieving CR (ELN criteria) (response duration 2 months to 3.3 years) and 2 (40%) PR or morphologic leukemia-free state; treatment-induced reduction in *IDH2* mutant allele burden was documented in half of the responders and response duration was generally less than 2 years; grade-5 differentiation syndrome was observed in 2 patients [37]. In a more recent preliminary report of 6 patients with MPN-BP/AP/CP from a phase-2 multicenter trial of enasidenib + ruxolotinib [47], an overall response rate of 40% included 3 patients with CR or CRi [47]. Of note, CR/CRi rate in *IDH*-mutated MPN-BP patients treated with HMA-Ven was 50% and not different than those with wild-type *IDH* [25]. Regardless, in both the HMA-Ven [25] and IDH2 inhibitor [37] treatment trials in MPN-BP/AP, morphologic and molecular evidence of chronic phase disease persisted despite the achievement of CR.

### Allogeneic stem cell transplant experience in advanced phase myelofibrosis: accelerated or blast phase disease

AHSCT currently constitutes the only treatment modality in chronic phase MF with the potential to prolong survival [48]. The same appears to be the case for MPN-BP [49] and MPN-AP [50]. A contemporary study of over 4000 patients with mostly chronic phase MF reported 3-year survival, relapse, and non-relapse mortality rates of 58%, 22%, and 29%, respectively [51]. The study also revealed a significant trend in terms of older age distribution (median 59.3 years) and utilization of matched unrelated donors (45.2%) in more recent times [51]. In another retrospective study of 35 patients with MPN-AP, receiving reduced-intensity AHSCT, a 5-year survival rate of 65% was reported vs. 64% in the comparator arm of patients with chronic phase disease; relapse rate was higher in patients transplanted with MPN-AP vs. chronic phase disease [50]. Of note, bridging chemotherapy was not utilized in the latter study, and there is currently no consensus on its use in MPN-AP [50].

The therapeutic value of AHSCT in MPN-BP was recently confirmed by the European Society for Blood and Marrow Transplantation (EBMT) registry-based analysis of 663 informative cases (median age 60 years; 61% males) [49]; median time from MPN-BP diagnosis to AHSCT was 4.4 months and median follow-up after AHSCT 5.2 years; pre-transplant treatment of MPN-BP included intensive chemotherapy in 49% of evaluable cases, HMA 6%, ruxolitinib 3% and other less intensive therapy in 33% [49]; type of donor included matched unrelated in 35% of patients, matched sibling in 28%, mismatched unrelated 18%, and mismatched related 9%; conditioning regimen was reduced-intensity in 65% and myeloablative in 35%; graft versus host (GvHD) prophylaxis included calcineurin inhibitor-based in 87%, post-transplant cyclophosphamide (PTCy) in 10% and T cell depletion in 69%; disease stage at the time of transplant was CR 45% and persistent disease 55%.

The above-introduced study by Orti et al. [49] reported a graft failure rate of 5.5% and median time to neutrophil and platelet engraftment of 18 and 20–23 days, respectively; CR in the first 100 days of AHSCT was documented in 76% of evaluable patients, and estimated 3- and 5-year post-transplant survivals were 36% and 32%, respectively; post-transplant survival was similar in the settings of matched sibling (37%) or matched unrelated (42%) donors and inferior in mismatched unrelated donors (25%). Importantly, the outcome was shown to be superior in the absence of active disease at the time of transplant (3-year survival 43% vs. 30%) while it was not affected by the intensity of the conditioning regimen; 3-year cumulative incidence of chronic GvHD was 35%, non-relapse mortality 24%, relapse 48%, progression-free survival 28%, and GvHD-free and relapse-free survival 18% [9]; in multivariable analysis, better performance status, absence of active disease at the time of transplant, and a more recent period of transplant independently predicted superior survival; the most common causes of death were disease progression and infection; post-transplant treatment included donor lymphocyte infusion (DLI; 20% of evaluable cases), mostly done because of mixed chimerism.

Table 3 outlines additional reports of AHSCT in the settings of MPN-BP or MPN-AP [49, 50, 52–59]. It should be noted that the second largest study listed by Kroger et al. (*N* = 422) [52] considered patients that were subsequently included in the abovementioned larger study by Orti et al. (*N* = 663) [49]. The additional informative value of the remaining reports listed in Table 3 is limited by a significantly smaller sample size ranging from an “*n*” of 14 to 177, further confounded by the heterogeneity of the study population, which included MPN-AP [50] and MDS/MPN-BP [54, 59], in some of the reports. Regardless, the possibility of long-term survival, in MPN-BP, with AHSCT was evident in all the informative studies with 5-year survival rates of 18% to 32% (Table 3).

**Table 3:** Summary of clinical studies on allogeneic hematopoietic stem cell transplant (AHSCT) for patients with blast or accelerated phase myeloproliferative neoplasms (MPN-BP/AP) (*References cited in the text*).

Study	MPN type/Median age	Pre-AHSCT chemotherapy	Disease status at AHSCT	Donor type	Conditioning regimen	GvHD GRFS	Relapse rate	Overall survival	Predictors of inferior survival
Orti et al. *AJH 2023* *N* = 663	MPN-BP 60 years	Intensive* 49% HMA 6% Ruxolitinib 3% Other 33%	CR 45%	Matched 65% MRD 28% MUD 35% MMUD 18% MMRD 9%	RIC 65% MAC 35%	1-year GRFS 29% 3-year GRFS 18%	3-year 48% 5-year 51%	3- year 36% 5-year 32%	Absence of CR Performance score AHSCT 2005-2010
Kroger et al. *BJH 2019* *N* = 422	MPN-BP 59 years	-	CR 43%	MRD 36% MUD 64%	MAC 34% RIC 66%	Acute 26% Chronic 48%	3-year 50%	3- year 33%	Unrelated donor Graft source (PB) Absence of CR Older age Performance score CMV+ Unfavorable karyotype Ex-vivo T cell depletion
Gupta et al. *Blood Advances* 2020 *N* = 177	MPN-BP 59 years	Intensive 57% HMA 4% None 6%	CR 57%	MRD 31% MUD 47% MMUD 15%	MAC 52% RIC 48%		5-year 66%	5-year 18%	Adverse karyotype *TP53* mutation Graft source (BM)
Cahu et al. *BMT 2014* *N* = 60	MPN-BP *n* = 43 MDS/MPN-BP *n* = 17 57 years	Intensive 78% HMA 5% None 12%	CR 43%	MRD 38% MUD 35% MMUD 27%	RIC 77% MAC 23%		3-year 68%	3-year 18%	Absence of CR Adverse karyotype
Lussana et al. *Haematologica* *2014* *N* = 57	MPN-BP 56 years	None 11%	CR 39%	MRD 46% MUD 50%	MAC 40% RIC 60%		53%	3-year 28%	
Alchalby et al. *BBMT 2014* *N* = 46	MPN-BP 55 years	Intensive 91%	CR 24%	MRD 37% MUD 57% MMUD 4%	RIC 57% MAC 43%	Acute 50% Chronic 63%	3-year 47%	3-year 33%	Absence of CR
Shah et al. *BJH 2021* *N* = 43	MPN-BP 59 years	Intensive 67% HMA 23% None 16%	CR 42%	MRD 40% MUD 60%	MAC 67% RIC 33%	Acute 46% Chronic 33%	43%	38%	AHSCT 2001-2009
Gagelmann et al. *Blood Advances* *2022* *N* = 35	MPN-AP 58 years	None	AP	MRD 23% MUD 63% MMUD 14%	RIC 100%		5-year 49% 5-year 31%	5-year 65%	*CALR/MPL* unmutated *RAS* mutation Performance score Age ≥57 years
Kennedy et al. *Blood 2013* *N* = 17	MPN-BP 59 years	Intensive 94%	CR 59%	MRD 70% MUD 24% MMUD 6%	RIC 81% MAC 13%	Acute 77% Chronic 53%	33%	2-year 15%	Age > 60 years Performance score BM blasts ≥50% Albumin <3.2 g/dl
Ruggiu et al. *BBMT 2020* *N* = 16	MPN-BP MDS-EB2 65 years	Intensive 42% HMA 40%	CR 69%	MRD 38% MUD 19% MMUD 19% Haplo 6% Cord 19%	MAC 52% RIC 48%			2- year 34%	
Ciuera et al. *BBMT 2010* *N* = 14	MPN-BP 59 years	Intensive 93%	CR 43%	MRD 57% MUD 29% Mismatched 14%	RIC 64% MAC 29%	Acute 50% Chronic 36%	29%	2-year 49%	

*Intensive- acute myeloid leukemia (AML) induction chemotherapy.

*HMA* hypomethylating agent, *CR* complete remission, *MRD* matched related donor, *MUD* matched unrelated donor, *MMUD* mismatched unrelated donor, *MMRD* mismatched related donor, *RIC* reduced-intensity conditioning, *MAC* myeloablative conditioning, *GRFS* GVHD relapse-free survival, *RI* relapse incidence, *RFS* relapse-free survival, *PB* Peripheral blood, *CMV* cytomegalovirus, *BM* bone marrow, *MPN* myeloproliferative neoplasm, *MPN-AP* accelerated phase MPN, *MPN-BP* blast phase MPN, *MDS* myelodysplastic syndrome, *MDS-EB2* MDS with excess blasts 2, *MDS/MPN* myelodysplastic/myeloproliferative.

### Our current treatment approach in accelerated or blast phase myeloproliferative neoplasm

We are acutely aware and forewarned about the dismal prognosis associated with MPN-BP. Accordingly, we prefer aggressively pushing toward AHSCT in intermediate or high-risk patients with chronic or accelerated phase MF before they progress into MPN-BP. In this regard, patient selection is facilitated by applying the karyotype- and mutation-enhanced international prognostic scoring system, version 2 (MIPSS*v2*) [60]. The latter utilizes nine components, including 5 genetic and 4 clinical [60]; the five genetic variables include very high risk (VHR; single or multiple abnormalities of -7, inv(3)/3q21, i(17q), 12p-/12p11.2, 11q-/11q23, autosomal trisomies other than +9 or +8)) karyotype (4 points), unfavorable (neither VHR or favorable; the latter being normal karyotype or sole abnormalities of 20q-, 13q-, +9, chromosome 1 abnormalities including 1q duplication, loss of Y chromosome or other sex chromosome abnormality) karyotype (3 points), ≥2 HMR mutations (3 points; *ASXL1*, *SRSF2*, *U2AF1Q157*), presence of one HMR mutation (2 points), absence of type 1/like *CALR* mutation (2 points); the four clinical variables in MIPSS*v2* include constitutional symptoms (2 points), severe anemia, defined by hemoglobin levels of <8 g/dl in women and <9 g/dl in men (2 points), moderate anemia, defined by hemoglobin levels of 8–9.9 g/dl in women and 9–10.9 g/dl in men (one point) and circulating blasts ≥2% (one point). MIPSS*v2* includes five risk categories: very high risk (≥9 points); high risk (5–8 points); intermediate risk (3–4 points); low risk (1–2 points); and very low risk (zero points); in patients aged 70 years or younger, the corresponding median survivals (10-year survival rates) were 1.8 years (<5%), 4.1 years (13%), 7.7 years (37%), 16.4 years (56%) and “median not reached” (92%).

**Fig. 1:**
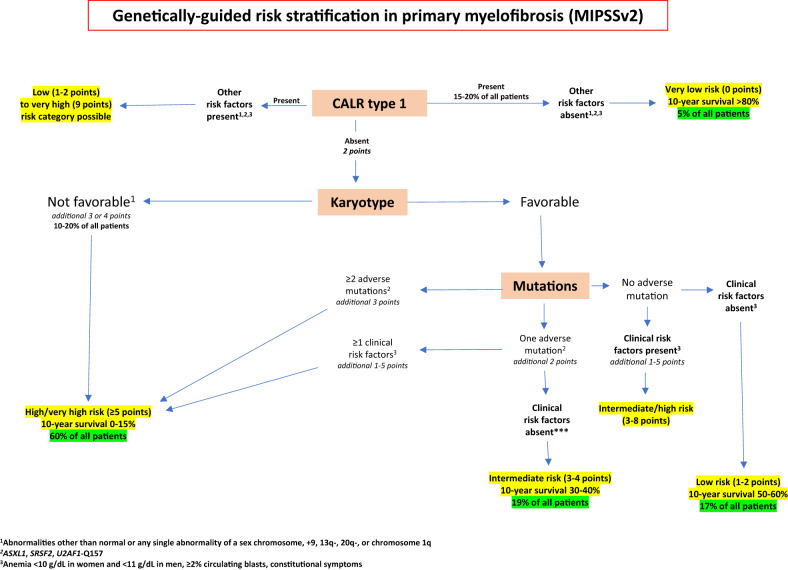
Current risk stratification in primary myelofibrosis.

Figure 1 provides a practical MIPSSv2-based risk stratification algorithm in MF that illustrates 10-year survival rates ranging from <5% (very high-risk disease) to >80% (very low-risk disease). The presence of type 1/like *CALR* mutation is a pre-requisite for “very low risk” disease (10-year survival estimate of 86–92%), which, in addition, requires the absence of high-risk mutations, unfavorable karyotype and adverse clinical features (Fig. 1). In the absence of of type 1/like *CALR* mutation, the most favorable risk category possible is “low risk” disease (10-year survival estimate of 50–56%), and such categorization also requires the absence of unfavorable karyotype, HMR mutations, and other clinical risk factors, as outlined above. Also, in the absence of type 1/like *CALR* mutation, the presence of either unfavorable karyotype, ≥2 HMR mutations, or one HMR mutation together with at least one clinical risk factor guarantees high (10-year survival estimate 10–13%) or very high (10-year survival estimate <5%) risk disease. Ten-year survival estimates in intermediate-risk patients range from 30 to 37%. Accordingly, based on MIPSSv2 risk assignment, AHSCT is advised sooner than later in high- or very high-risk disease, while it is reasonable to defer the procedure in chronic phase MF patients with low- or very low-risk disease; on the other hand, therapeutic decision making in the intermediate-risk patient requires an individualized treatment approach that considers age, performance status, availability of experimental drug therapy, and the wishes of the patient and their families.

**Fig. 2:**
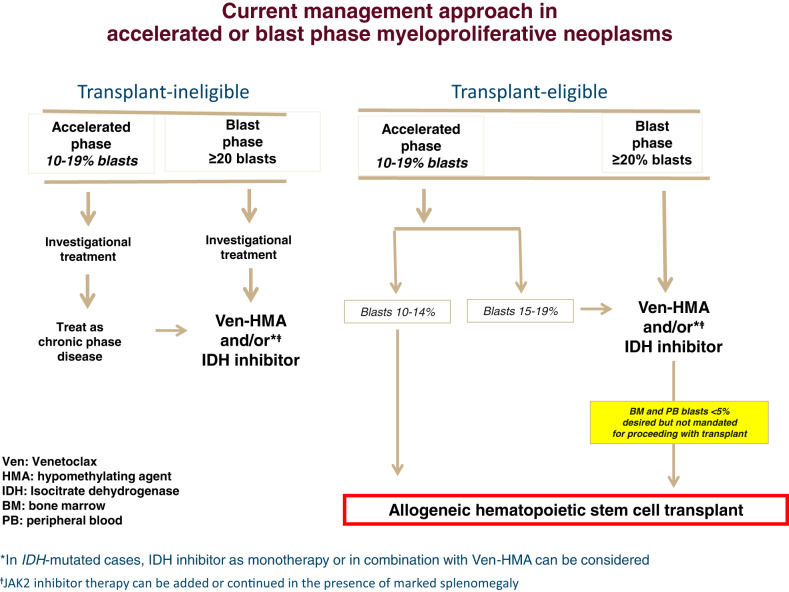
Current treatment algorithm for accelerated or blast phase myeloproliferative neoplasm.

We highly recommend pursuing AHSCT as soon as possible in the setting of both MPN-AP and MPN-BP; this is because it is currently unlikely that chemotherapy alone, either in the context of investigational drug therapy or outside of a protocol setting, would guarantee long-term survival. Patients who are not eligible for transplants might be best served by participation in a clinical trial (Fig. 2) since durable responses are unlikely with currently available drugs (Table 2). Otherwise, a combination of venetoclax with HMA or, in *IDH*-mutated cases, IDH inhibitor monotherapy or combination with HMA ± Ven might provide a short-term survival advantage over supportive care (Table 2; Fig. 2). In this regard, there is no convincing evidence that anything else would perform better than HMA-Ven and achievement of a higher CR rate from a particular induction regimen does not necessarily translate into longer survival. In transplant-eligible patients, the key initial consideration is whether or not bridging chemotherapy is necessary and, if so, what the optimal treatment regimen might be. There are no controlled prospective studies to inform decision-making in this regard, and our current practice is based on our interpretation of available information from retrospective observations.

In a retrospective study of 35 patients with MPN-AP, receiving reduced-intensity AHSCT without bridging chemotherapy, a 5-year survival rate of 65% was reported vs. 64% in the comparator arm of patients with chronic phase disease; the relapse rate was higher in patients transplanted with MPN-AP vs. chronic phase disease [50]. Accordingly, in MPN-AP, we currently exercise an individualized approach in the implementation of bridging chemotherapy based on bone marrow/circulating blast burden and the likelihood of response to HMA-Ven (Fig. 2). In other words, we are inclined to proceed directly to transplant in patients with lower levels of blast burden and in those who are unlikely to respond to Ven-HMA; otherwise, it is reasonable to implement one or two cycles of Ven-HMA or, in *IDH*-mutated cases, IDH inhibitor monotherapy or combination with HMA ± Ven, prior to transplant (Fig. 2). It should also be noted that pre-transplant ruxolitinib therapy is increasingly being used, in both chronic and accelerated phase MF, in order to reduce spleen burden in patients with marked splenomegaly and facilitate engraftment [17].

At present, we favor the pre-transplant implementation of bridging chemotherapy in MPN-BP, with the objective of attaining CR/CRi or marrow CR (Fig. 2). In the aforementioned study by Orti et al. [49], the absence of active disease at the time of transplant was associated with a higher 3-year survival rate (43% vs. 30%). Not being in CR was also an independent risk factor for post-transplant survival in another study with a broader group of patients with secondary AML, including those with MPN-BP and transformed chronic myelomonocytic leukemia or myelodysplastic syndrome [52]. In our own experience with HMA-Ven induction in MPN-BP, the presence or absence of complex/monosomal karyotype and *N/KRAS* mutations, as opposed to disease status at transplant, appeared to be more important in predicting post-transplant survival [25]. Others have made similar observations [53]. In regards to patients not achieving optimal response to HMA-Ven or, in *IDH*-mutated cases, to IDH inhibitor monotherapy or combination with HMA ± Ven, we favor proceeding with transplant sooner rather than attempting additional salvage therapy in search of CR; in a recent randomized study of patients with relapsed/refractory AML, post-transplant survival was not favorably affected by additional intensive chemotherapy with the objective to attain CR, as opposed to sequential conditioning followed directly by AHSCT [61]. It is also to be noted that a sizable proportion of patients (30%) with active disease at the time of transplant, in the study by Orti et al. [49], were successfully salvaged by AHSCT, supporting the argument that earlier institutions of transplant with active disease, in certain circumstances, might be preferred over delaying AHSCT in search of CR and putting patients at risk for complications associated with additional salvage chemotherapy (Fig. 2).

### Concluding remarks

Considering the current consensus that AHSCT is indispensable for long-term survival in MPN-BP, the focus going forward should be on measures that can be undertaken in order to optimize post-transplant survival and identify patients who are unlikely to benefit from the procedure, such as those with multi-hit *TP53* mutations [62]. The overarching principle in managing patients with myelofibrosis is to avoid delay in aggressively pursuing transplant in patients with intermediate/high-risk chronic phase or accelerated phase disease, based on the inaccurate assumption of survival benefit from JAK2 inhibitor therapy [63]. Pre-transplant bridging chemotherapy is currently not indicated in chronic phase MPN, and its value in MPN-AP is controversial and individually approached [50]. HMA-Ven is currently the standard induction therapy for patients with MPN-BP, most of whom would have been on JAK2 inhibitor therapy during disease progression [25]; we have no objections to the use of alternative induction regimens and, in the presence of *IDH* mutations, single-agent therapy with an IDH inhibitor might be less toxic than HMA-Ven and adequate enough as a bridge toward AHSCT [37]. The potential to achieve an even higher blast clearance rate in *IDH*-mutated patients with MPN-BP, using a triple combination of IDH inhibitor, Ven ± HMA has been suggested by recently published experience in the setting of *IDH1*-mutated myeloid malignancies (*N* = 31) with a composite complete remission rate of 83–90% [64]; IDH1 mutation clearance and MRD-negative status were documented in the majority of patients. Similarly, a recent report suggested that AML with erythroid or megakaryocytic differentiation depend on BCL-XL more than they do on BCL-2, thus providing another therapeutic target for future studies, especially in patients resistant to venetoclax-based therapies [65].

Persistence of active disease after induction chemotherapy for MPN-BP might be a marker of aggressive disease biology that is not necessarily modified by repeated courses of salvage chemotherapy, which, instead, might result in treatment-related complications with the potential to compromise transplant eligibility; accordingly, we prefer proceeding with transplant earlier than dictated by residual disease, where additional salvage therapy might not have an overall impact on survival [61]. As for additional considerations regarding donor selection, conditioning, and management of relapse and poor graft function, the protocol we follow is not significantly different from that applied to patients with chronic phase disease [66]. Future research efforts should include prospective controlled studies targeting optimal conditioning regimens, sensitive methods of measurable residual disease monitoring, standardization of intervention points for donor lymphocyte infusions, and innovative pre-emptive therapy to minimize post-transplant relapse [67].
